# Alternative Splicing: A Potential Source of Functional Innovation in the Eukaryotic Genome

**DOI:** 10.1155/2012/596274

**Published:** 2012-07-02

**Authors:** Lu Chen, Jaime M. Tovar-Corona, Araxi O. Urrutia

**Affiliations:** Department of Biology and Biochemistry, University of Bath, Bath BA2 7AY, UK

## Abstract

Alternative splicing (AS) is a common posttranscriptional process in eukaryotic organisms, by which multiple distinct functional transcripts are produced from a single gene. The release of the human genome draft revealed a much smaller number of genes than anticipated. Because of its potential role in expanding protein diversity, interest in alternative splicing has been increasing over the last decade. Although recent studies have shown that 94% human multiexon genes undergo AS, evolution of AS and thus its potential role in functional innovation in eukaryotic genomes remain largely unexplored. Here we review available evidence regarding the evolution of AS prevalence and functional role. In addition we stress the need to correct for the strong effect of transcript coverage in AS detection and set out a strategy to ultimately elucidate the extent of the role of AS in functional innovation on a genomic scale.

## 1. Introduction

The first draft of the human genome sequence [1, 2] was unveiled in February 2001 and surprisingly it was shown to contain ~23000 genes, only a fraction of the numbers of genes originally predicted [3]. To put this into perspective, there are ~20,000 genes in the genome of the nematode *C. elegans*. The lack of an association between gene number and organismal complexity has resulted in an increased interest in alternative splicing (AS) given it has been proposed to be a major factor in expanding the regulatory and functional complexity, protein diversity, and organismal complexity of higher eukaryotes [4–6]. However, despite the best efforts of many research groups we still understand very little about the actual role played by AS in the evolution of functional innovation—here understood as the appearance of novel functional transcripts—underpinning the increased organismal complexity observed.

Alternative splicing is a posttranscriptional process in eukaryotic organisms by which multiple distinct transcripts are produced from a single gene [4]. Previous studies using high-throughput sequencing technology have reported that up to 92%~94% of human multiexon genes undergo AS [7, 8], often in a tissue/developmental stage-specific manner [7, 9]. With the development and constant improvement of whole genome transcription profiling and bioinformatics algorithms, the ubiquity of AS in the mammalian genome began to become clear. The concept of one gene-one protein gave way as evidence mounted for the high percentage of AS incidence in nonhuman species [7, 8], such as fruit fly [10], *Arabidopsis* [11] and other eukaryotes [5]. Despite the advances in our understanding and characterisation of AS several questions remain unanswered. First, the large difference in transcript coverage between species has hampered direct comparisons of the prevalence of alternative splicing in different species [6]. Secondly, even if comparable AS estimates between species could be obtained, it is unclear to what extent any changes in AS prevalence along evolution have contributed to overall protein diversity or rather reflect splicing noise. Finally, we understand very little about how AS has evolved through time and how this is related to functional parameters of genes. Here we review how alternative is regulated and recent progress in our understanding of the evolution of alternative splicing.

## 2. Alternative Splicing and Its Regulation

In 1977, Chow et al. [12–15] reported that 5′ and 3′ terminal sequences of several adenovirus 2 (Ad2) mRNAs varied, implying a new mechanism for the generation of several distinct mRNAs. Following this study, alternative splicing was also found in the gene encoding thyroid hormone calcitonin in mammalian cells. Subsequent studies revealed that many other genes were also able to generate more than one transcript by cuttingout different sections from its coding regions (reviewed in [4, 16]).

**Figure 1:**
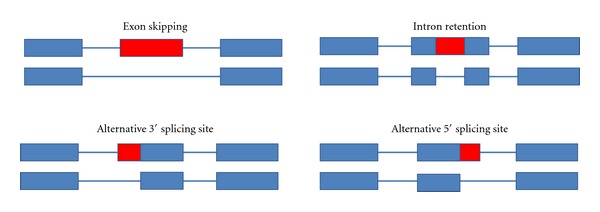
Different types of alternative splicing. The blue boxes are constitutive exons and alternatively spliced regions in red. Introns are represented by straight lines between boxes. Four types of common splicing events were identified: (1) exon skipping (2) intron retention (3) alternative 5′ splicing site (5′ss), and (4) alternative 3′ splicing site (3′ss).

Depending on the location of the exonic segments cut-out-or if introns are left in, splicing events can be classified into four basic types (Figure 1). These four major modes of splicing are (1) exon skipping (2) intron retention (3) alternative 5′ splicing site (5′ss), and (4) alternative 3′ splicing site (3′ss) [22, 23]. In addition, mutually exclusive exons, alternative initiation, and alternative polyadenylation provide two other mechanisms for generating various transcript isoforms. Moreover, different types of alternative splicing can occur in a combinatorial manner and one exon may be subject to more than one AS mode, for example, 5′ss and 3′ss at the same time (Figure 1). Prevalence of each type of AS has been found to vary between different taxa. Several studies have shown that exon skipping is common in metazoan genomes [24] whereas intron retention is the most common type of AS among plants [25] and fungi [26].

Alternative splicing is tightly regulated by cis elements as well as transacting factors that bind to these cis elements. Transacting factors, mainly RNA-binding proteins, modulate the activity of the spliceosome and cis elements such as exonic splicing enhancers (ESEs), exonic splicing silencers (ESSs), intronic splicing enhancers (ISEs), and intronic splicing silencers (ISSs). Canonical mechanism of AS suggests that serine/arginine-rich (SR) proteins typically bind to ESEs, whereas heterogeneous nuclear ribonucleoproteins (hnRNP) tend to bind to ESSs or ISSs [27]. Given the crucial roles of these regulators in the splicing machinery, the cis and transacting mutations, which disrupt the splicing code, are known to cause disease (reviewed in [28–30]). It has been estimated that 15–60% of mutations cause disease by affecting the splicing pattern of genes ([31] and reviewed in [30]). Moreover, AS has also been shown to be regulated without the involvement of auxiliary splicing factors [32] and AS may be also combined with other posttranscriptional events such as the use of multiple internal translation initiation sites, RNA editing, mRNA decay, and microRNA binding and other noncoding RNAs [33, 34], suggesting the existence of additional noncanonical mechanism of AS that are yet to be identified [35]. 

Recently, a direct role of histone modifications in alternative splicing has been reported, in which histone modification (H3-K27m3) affects the splicing outcome by influencing the recruitment of splicing regulators via a chromatin-binding protein in a number of human genes such as *FGFR2,TPM2,TPM1* and *PKM2* [36]. Moreover, it has been reported that CTCF-promoted RNA polymerase II pausing links DNA methylation to splicing, providing the first evidence of developmental regulation of splicing outcome through heritable epigenetic marks [37]. Additionally, non-coding RNAs also have emerged as key determinants of alternative splicing patterns [34]. Therefore these findings reveal an additional epigenetic layer in the regulation of transcription and alternative splicing [38]. Genomewide genetic and epigenetic studies, therefore, have been proposed in at least 100 specific blood cell types [39], which will provide high quality reference epigenomes (using DNA methylation and histone marks assays) with detailed genetic and transcriptome data (whole genome sequencing, RNA-Seq, and miRNA-Seq), providing us with an opportunity to assess the genomewide influence of epigenetic factors in the regulation of AS in specific blood cell types. We are expecting the rise of comparative epigenetics will provide different perspective of the evolution of transcriptome.

## 3. Identification of Alternative Splicing Events

Alternative splicing is difficult to estimate from genomic parameters alone [40]. A number of regulatory motifs for AS have been uncovered but the presence of known alternative splicing motifs does not guarantee that a gene is actually alternatively spliced [40]. Thus, alternative splicing patterns are generally assessed from examining transcript data. For any gene of interest, alternative splicing events can be identified by using reverse transcription polymerase chain reaction (RT-PCR) conducted on a complementary DNA (cDNA) library. Over the last decade, as high-throughput transcriptome technologies have improved, it has become possible to assess alternative splicing patterns on a genomewide scale. Three main sources of transcriptome data have been used to assess splicing patterns: expressed sequence tags (ESTs), splice-junction microarrays, and RNA sequencing (RNA-Seq). 

The first wave of genomewide transcriptome analysis consisted in direct sequencing cDNA and ESTs carried out at large scale [41], which allowed alternative splicing events to be identified by aligning cDNA/EST sequences to the reference genome. ESTs are 200–800 nucleotide bases in length, unedited, randomly selected single-pass sequence reads derived from cDNA libraries [42]. Currently, there are eight million ESTs for human, including about one million sequences from cancer tissues, and about 71 million ESTs for around 2000 species in dbEST [43]. However, ESTs are based on low-throughput Sanger sequencing and are aggregated over a wide range of tissues, developmental states, and diseases using widely different levels of sensitivity.

More recently, splice-junction microarrays and RNA-Seq have been increasingly used to quantitatively analyse alternative splicing events. Splicing microarrays target specific exons or exon-exon junctions with oligonucleotide probes. The fluorescent intensities of individual probes reflect the relative usage of alternatively splicing exons in different tissues and cell lines [44]. High-density splice-junction microarrays are a cost-effective way to assay previously known exons and AS events with low false positive rate. The disadvantage is that it requires prior knowledge of existing AS variants and gene structures. More importantly unlike RNA-Seq and EST, microarrays do not provide additional sequence information.

RNA-Seq has emerged as a powerful technology for transcriptome analysis due to its ability to produce millions of short sequence reads [45–47]. RNA-Seq experiments provide in-depth information on the transcriptional landscape [45]. The ever-increasing accumulation of high-throughput data will continue to provide ever richer opportunities to investigate further aspects of AS such as low-frequency AS events as well as tissue-specific and/or development-specific AS events [7, 8, 47–49]. Earlier datasets consist of RNA read sequences of 50 bp or less, limiting the information about combinations of AS events in a single transcript but it is likely that the length of short reads will continue to increase over the next decade. With the increasing capacity of next-generation sequencing (RNA-Seq) the study of alternative spicing is likely to undergo a revolution [50]. The higher depth of sequencing of transcriptomes in human and other species has increased our understanding of the occurrence of AS event and AS expression patterns in different tissues [7, 51], developmental stages [10]. 

Transcript assembly of sequence-based technologies, such as ESTs and RNA-Seq, can use either align-then-assemble or assemble-then-align, depending on the quality of reference genome and sequence data [47]. An algorithm can be employed to detect AS event by comparing different transcripts. However, detecting AS isoforms, as opposed to single AS event, is still challenging because short sequences provide little information in terms of the combination of exons. Several applications have been developed for transcript assembly and AS isoform detection, different strategies and comparison of these applications have been reviewed previously [47].

## 4. Prevalence of Alternative Splicing across Eukaryotic Genomes

Initial whole genome analyses suggested that 5%–30% of human genes were alternatively spliced (reviewed in [6, 16]). EST-based AS databases identify AS events in 40–60% of human genes [5, 52, 53]; however, recently this number has been revised over and over with the latest estimates showing that up to 94% of human multiexon genes produce more than one transcript through alternative splicing [7, 8, 16]. Understanding how alternative splicing has changed over time could provide insights as to how alternative splicing has impacted on transcript and protein diversity and phenotype evolution [6]. In fungi, AS is thought to be rare due to the low number of exons in yeast [23]. In plants it has been estimated that around 20% of genes undergo AS based on EST data [25], a recent study using RNA-Seq, however, suggests that at least approximately 42% of intron-containing genes in *Arabidopsis* are alternatively spliced [11]. We are expecting significantly higher percentages of AS occurrence will be discovered from various eukaryotes given the in-depth studies of transcriptome using next-generation sequencing such as RNA-Seq are ongoing. A few studies have attempted to compare AS prevalence among different taxa with animals generally reported to have higher AS incidence than plants [16] and vertebrates having a higher AS incidence than invertebrates [24]. However, these studies are either based on limited data or failed to correct for differences in transcript coverage [6].

**Figure 2:**
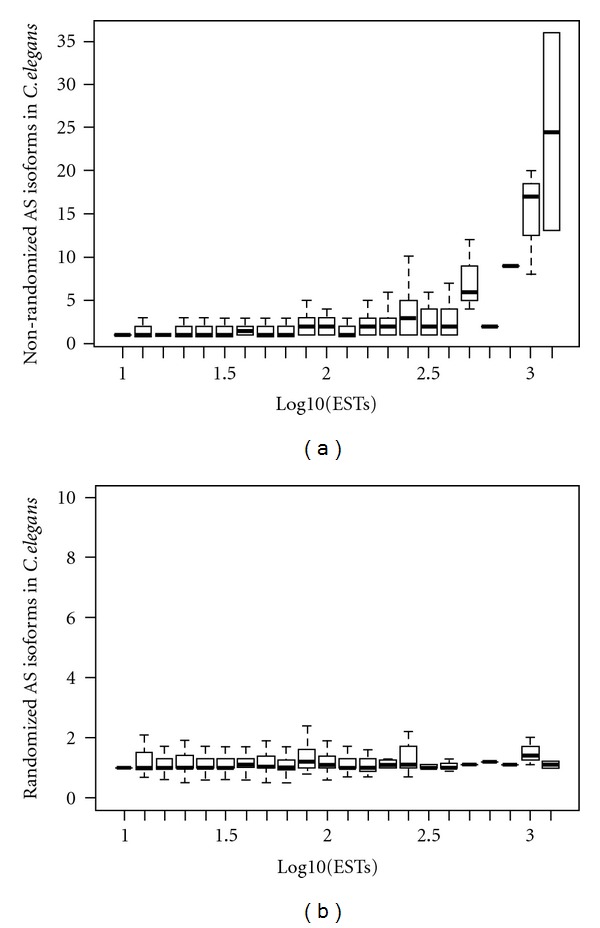
Total transcript number influences AS detection but bias can be corrected by using a sampling method. AS detection in genes divided by transcript coverage for the nematode (a and b) using the full transcript dataset (a) or a random sampling method (b).

There are a number of databases that provide AS data for multiple species [5, 52–54]. However, these existing resources are primarily focused on animal species and have poor coverage for protist, fungal, and plant genomes thus making it difficult to compare divergent taxa. Most importantly, none of these resources take into account the well-documented effects of differential transcript coverage across genes within and between species which greatly influences AS detection rates [6, 24, 55, 56]. Random sampling has been used [24] and shown to minimize the bias of transcript coverage (Figure 2). We expect that similar strategies will be employed in future comparative AS data resources.

## 5. Is Alternative Splicing Functional or Mostly Just Noise?

If an increase in AS levels in vertebrate species compared to invertebrates is confirmed, given the limitations of current proteomics resources, it is hard to assess the extent to which alternatively spliced transcripts are translated into an expanded proteome. The evolution of many phenotypes that we most associate with human being such as longer lifespan, encephalization, or even increased complexity have been accompanied by sharp reductions in effective population size, possibly explaining the proliferation of a variety of genomic features in more complex organisms ([57] but see [58]). Therefore, it is possible that increased AS through evolution results from aberrant splicing and therefore it does not play any functional role [59–61]. If alternative splicing has increased along the phylogenetic tree and it is indeed functional, we can expect the following.

**Figure 3:**
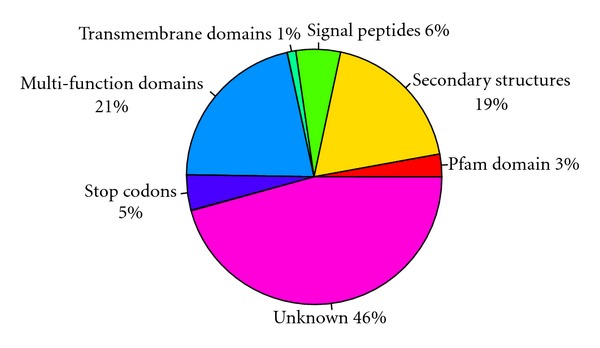
Percentage of AS areas containing identifiable functional domains, secondary structures, and stop codons in human. Functional components were identified using InterProScan which contains 14 applications for the prediction of protein domains [17], including Pfam for the prediction of protein domains [18], SignalP 3.0 for signal peptide predictions [19], and TMHMM [20] for the predictions of transmembrane domains. PSORT II [21] was used to identify the likely subcellular localization of protein products. Secondary protein structures were predicted by CLC Main Workbench 5.7, which is based on extracted protein sequences from the protein databank (http://www.rcsb.org/pdb/).

- Transcripts should have a low incidence of premature stop codons which would render them vulnerable to nonsense mediated decay. Between 4% and 35% of AS human transcripts have been found to contain a premature termination codon in human and mouse transcripts [62, 63]. These transcripts have been found to be enriched in nonconserved exons likely to cause frame shifts [64]. It is unknown whether the proportion of premature stop codon containing AS transcripts has changed along the phylogenetic tree.
- It has been proposed that most low copy number alternative isoforms produced in human cells are likely to be nonfunctional [65, 66]. A recent study has shown that although cancer-specific alternative-splicing variants can be found, these events are mostly found as single-copy events and thus unlikely to contribute to the core cancer transcriptome [67].
- Conservation of alternative-splicing events along evolution can be taken as an indicator of their functional role. Conservation levels of AS have been studied in many species. The estimation ranges from 11% to 67% between human and mouse [68–70]. Notably, major AS forms tend to be have higher conservation levels compared to minor forms. On the other hand, the conserved AS forms vary among different AS; for example, exon skipping between *C. elegans* and *C. briggsae *has shown more than 81% conservation level, compared to 28% for intron retention [71, 72].
- Presence of identifiable functional domains in AS areas may also be an indicator of functional relevance for AS transcripts [67]. To our best knowledge there are no reports of the prevalence of functional domains in AS areas in model species. To examine the presence of functional domains in AS transcripts, we compiled a set of 267,996 AS events obtained from the analysis of 8,315,254 ESTs from normal human tissues. We found that about 50% of AS areas in human contain known functional components using InterProScan [17] which contains 14 applications for the prediction of protein domains (Figure 3, see methods in [67]), suggesting a possible functional role for AS. The extent of the variations in the prevalence of functional domains among AS areas between species remains to be explored but would provide additional insights on the evolution of AS.

Taken together above observations suggest that although alternative splicing-events are indeed conserved throughout evolution a significant proportion are not and some may result from noisy transcript splicing not contributing to the protein pool. However, until further studies using comparable AS indexes it will be impossible to estimate the extent to which increases in AS levels along the phylogenetic tree have impacted on the pool of functional transcripts.

## 6. Alternative Splicing and Gene Duplication

Gene duplication (GD) is considered a prime source of functional innovation in the genome. Newly duplicated genes can evolve functional divergence [73], and it is thought to be key in driving the evolution of developmental and morphological complexity in vertebrates [74]. Alternative splicing, as a prevalent mechanism that also increases protein diversity, has been proposed as a potential player in the evolution of eukaryotes [4, 6]. By examining the relationship between gene duplication and alternative splicing we can better understand the extent to which both mechanisms are equivalent means for protein diversification. Several studies have reported a negative correlation between AS and gene family size in human and mouse [6, 65, 75, 76] and worm [71, 77] (Table 1). It is easy to lead to a conclusion that AS and GD are interchangeable and there is a universal negative correlation from worm to human. However, the relationship between the two variables is marginal at best and it is not consistent when including singleton genes which have a lower AS level compared to multigene families [76, 78, 79]. Jin et al. [76] suggested that singletons have more evolutionary constriction than duplicates which hampers their AS isoform gain Consistent with this hypothesis, Lin et al. [78] found that singletons differ from multigene families in several aspects suggesting that they have differing evolutionary paths. Even if we focus on multigene families only, a negative correlation between AS and gene family size may be explained or byproduct of AS and gene family size covariance with other factors. For example, gene age and biased duplication have been proposed to be the explanation [79]. This study has cast doubt over the relationship between AS and GD and it may indeed provide support to the suggestion that AS and GD have little or no equivalence concerning effects on protein sequence, structure, and function [80]. As most studies have examined a small number of model species it is difficult to assess the extent of the link between AS and GD. In addition, the snapshot approach of comparing GFS and AS in a single genome might hide the true relationship between AS and GFS.

## 7. Alternative Splicing's Contribution to Functional Innovation

Alternative splicing has been hailed as the missing source of information in the genome accounting for the evolution of higher complexity despite the near static gene number in metazoans over the last 800 million years. Wegmann et al. [81] found that width of gene expression is positively correlated to the number of new transcript isoforms and proposed that the increase of gene expression breadth is essential for acquiring new transcript isoforms, which could be maintained by a new form of balancing selection. Moreover, experimental and bioinformatics analyses have shown that AS can generate a variety of functional mRNAs and protein products, displaying distinct stability properties, subcellular localization, and function [9] as well as in specific stages in cell differentiation [82], sex differentiation [83, 84], and development [9].

**Figure 4:**
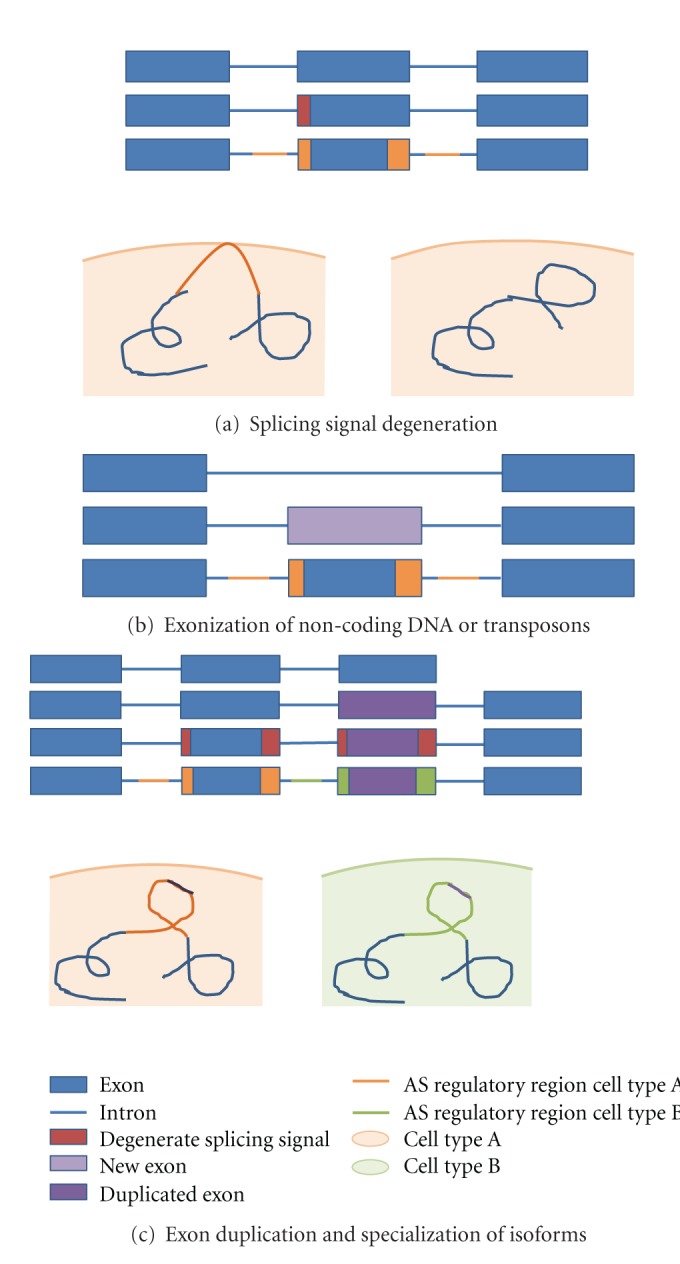
Novel AS variants can take on specialised or novel roles. Novel splicing variants can arise from (a) mutations in the exon recognition site of a constitutive exon and subsequent acquisition of AS regulatory elements. (b) Exonization of introns or intron regions or transposable elements with subsequent acquisition of AS regulatory regions. Novel proteins may interact with different proteins or localise in different subcellular regions. (c) Exon duplication and subsequent specialization functional domains and AS regulatory regions. Resulting specialised proteins may take on partial roles relevant in different cell types or developmental stages or result in novel interactions and functions.

Single-gene studies have provided examples where alternative splicing can lead to functional innovation before any events of gene duplication have taken place. One such example is that of Troponin I (TnI), which plays a key role in muscle contraction. In the vertebrate genome, TnI exists in three copies each expressed in a different muscle type (skeletal, fast and slow, and cardiac). In *Ciona*, one of the closest relatives of vertebrates TnI is present as a single gene. Interestingly, however, the *Ciona* gene produces three distinct alternatively spliced isoforms, each found to resemble the expression profile of one of the vertebrate genes suggesting that the specialisation of the TnI proteins to function in each muscle type preceded gene duplication events [85]. This pattern of alternative splice variants in ancestrally single genes resembling expression profiles of genes later duplicated has also been found in synapsin-2 genes in tetrapods [86] and *MITF* genes in teleost fish species [87, 88]. These examples suggest that alternative splicing can be a mechanism for functional innovation preceding events of gene duplication through one of the three possible paths (Figure 4).

Genes may also further gain alternative splicing and regulation after duplication along with the complexity of the organ systems after the divergence of protochordates and vertebrates. Comparison between transcriptional factors *Pax* genes in vertebrates and amphioxus has shown that at least 52 reported alternative-splicing events in vertebrates compared to 23 events in amphioxus [89]. Furthermore, vertebrate *Pax* genes have maintained most of their ancestral functions and also expanded their expression [90]. Novel alternative splicing of *Pax* genes has been shown to modify the functional domain content (e.g., DNA binding) and transactivation capacities of the resulting protein products [89]. For example, a novel alternative transcript of *Pax3* can transactivate a cMET reporter construct in mouse [91]. These additional isoforms of *Pax3* have been proposed to play a functional role in the acquisition of new roles at neural plate in vertebrates [89]. Similarly, vertebrate-specific AS events of exon 5a in *Pax4 *and *Pax6 *have been linked to functional roles in the development of vertebrate eye [89, 92]. Therefore, it is reasonable to propose the hypothesis that, besides gene duplication, alternative splicing plays important roles in acquiring novel functions contributing to the complexity of the organ systems after the divergence of protochordates and vertebrates [93]. The potential roles of the increasing prevalence of AS in vertebrates in functional innovation will be largely explored in more gene families or genomewide level in the future, which will further our understanding of how AS contributes to functional innovation.

## 8. Conclusion

Here we have reviewed evidence from genomewide studies as well as possible avenues for future comparatives studies for the potential of alternative splicing as a source of functional innovation during the evolution of the eukaryotic genome. While it is now clear that AS is prevalent in the human genome, obstacles still remain in the assessment how alternative splicing has evolved through time. The main obstacle lies in that while most other genomic features can be directly measured or estimated from genomic sequences alone, no accurate estimates of alternative splicing can be obtained from genomic sequence analysis. The reliance in transcript sequences availability to measure AS together with the strong bias brought by unequal transcript coverage has hampered the genomewide assessment of AS in all but a few model species and makes difficult any direct comparison between species. This has slowed down the study of how alternative splicing has evolved over time, how AS is regulated, and how it may relate to other genomic features and most crucially to phenotype. The ever-increasing transcript profiling for many more species combined with the use of comparable index estimates will allow addressing a number of evolutionary questions regarding the evolution of AS and its implications for the evolution of transcript diversity and functional innovation. 

## Figures and Tables

**Table 1:** Summary for the relationship between AS and GFS.

Species	Data	Alternative splicing	Orthology	Bias control	Correlation	Reference
Human	Ensembl	ASD's AltSplice database	BLSATP	Exons, EST coverage, gene family size, isoform count	Negative correlation	[75]
	NCBI, UCSC	GeneSplicer program	EnsMart	Remove garbage EST, EST coverage,	Negative correlation	[65]
	H-InvDB 5.0	H-InvDB 5.0	BLAST		Positive correlation when includes all gene families. Negative correlation within multigene families	[76]
Mouse	Ensembl	ASD's AltSplice database	BLSATP	Exons, EST coverage, gene family size, isoform count	Negative correlation	[75]
	NCBI, UCSC	GeneSplicer program	EnsMart	Remove garbage EST, EST coverage,	Negative correlation	[65]
	Riken's FANTOM3	Riken's FANTOM3	BLAST		Positive correlation when includes all gene families. Negative correlation within multigene families	[76]
*C.* *elegans *	WormPep	WormPep	BLAST		Lower AS occurrence in multigene families	[77]
Rice	TIGR 4.0	PASA program	BLASTP	Remove genes that lack transcript evidence	Multigene families have significantly higher AS incidence than singletons	[78]
Arabidopsis	TAIR7	TAIR7	TAIR7		Multigene families have significantly higher AS incidence than singletons	[78]
